# The Gut–Muscle Axis in Polycystic Ovary Syndrome: Proposed Mechanistic Roles of Intestinal Permeability, Bile Acid Signalling, and Skeletal Muscle Dysfunction

**DOI:** 10.3390/metabo16100758

**Published:** 2026-10-09

**Authors:** Aiswarya Prasad, Ashwini Kishan, Sreedhish Keezhadath

**Affiliations:** 1 Department of Physiology, KS Hegde Medical Academy (KSHEMA), Nitte (Deemed to be University), Mangalore 575018, India; draiswarya.p@nitte.edu.in; 2 Department of Orthopaedics, KS Hegde Medical Academy (KSHEMA), Nitte (Deemed to be University), Mangalore 575018, India; drsreedhish.k@nitte.edu.in

**Keywords:** polycystic ovary syndrome, gut–muscle axis, intestinal permeability, bile acids, FXR, TGR5, skeletal muscle dysfunction, sarcopenic obesity

## Abstract

**Highlights:**

**What are the main findings?**

- Women with PCOS show gut barrier dysfunction (elevated serum zonulin) and a distinct serum bile acid profile, with the latter currently described in a preprint awaiting peer review, that may converge on skeletal muscle insulin resistance.
- In one DXA-based case–control study using a study-specific definition, 53% of women with PCOS met criteria for sarcopenic obesity, with percentage appendicular skeletal muscle of 23.8% vs. 30.4% in controls.

**What are the implications of the main findings?**

- A proposed dual-axis model (LPS–TLR4 inflammation plus bile acid–FXR/TGR5 signalling) predicts that gut-derived signals contribute to muscle impairment beyond inflammation alone.
- Combined microbiome-, bile-acid-, and exercise-based dual-axis strategies warrant testing to preserve muscle quality in PCOS.

**Abstract:**

Polycystic ovary syndrome is a heterogeneous endocrine and metabolic disorder diagnosed when at least two of three features—chronic anovulation, hyperandrogenism, and polycystic ovarian morphology—are present, according to the Rotterdam criteria endorsed by the 2023 International Evidence-Based Guideline. Its metabolic burden extends well beyond reproduction. Insulin resistance, low-grade systemic inflammation, and central adiposity are common, and recent work links the composition of the gut microbiota and the integrity of the intestinal barrier to each of these features, the association between dysbiosis and the pathogenesis of the syndrome having been reviewed in detail. The same gut-related disturbances are now being connected to a second, less recognized consequence of the syndrome: impaired skeletal muscle health. In one cohort, an increased prevalence of sarcopenic obesity has been reported in PCOS despite the anabolic potential of androgen excess, and this review proposes that the two observations are mechanistically linked. Two candidate pathways connect the gut to muscle in PCOS. The first runs from increased intestinal permeability to circulating lipopolysaccharide, which activates Toll-like receptor 4 and a cascade of pro-inflammatory cytokines that blunt insulin signalling in skeletal myocytes. The second runs through bile acids: PCOS is characterized by a distinct circulating bile acid profile, currently described in a preprint that has not undergone peer review, while peer-reviewed profiling has confirmed altered circulating bile acid signatures and links to hyperandrogenism in women with PCOS. Signalling through the farnesoid X receptor and Takeda G protein-coupled receptor 5 has been shown to regulate muscle insulin sensitivity, mitochondrial oxidative capacity, and protein turnover in experimental systems, although direct demonstration in human PCOS muscle is lacking. Both pathways are predicted to converge on the IRS-1–PI3K–Akt–mTORC1 axis, the signalling hub through which muscle cells normally translate insulin into glucose uptake and protein synthesis. After reviewing the evidence for gut barrier dysfunction and skeletal muscle impairment in PCOS, we propose a dual-axis model linking intestinal permeability, bile acid signalling, and sarcopenic obesity. We then consider the therapeutic potential of interventions targeting the microbiota, bile acid receptors, inflammation, and muscle function, and outline the study designs that could test the model directly.

## 1. Introduction

Polycystic ovary syndrome affects 8–13% of women of reproductive age, and it has become clear that the condition does not end at the menopause transition [1]. Alongside the classical reproductive features chronic anovulation, hyperandrogenism, and polycystic ovarian morphology—of which any two suffice for diagnosis under the Rotterdam criteria—affected women carry a metabolic burden of insulin resistance, dyslipidemia, central adiposity, and low-grade inflammation, raising the lifetime risk of type 2 diabetes, non-alcoholic fatty liver disease, and cardiovascular disease by roughly two- to fourfold [2,3].

At first glance, the muscle phenotype of PCOS appears paradoxical: androgen excess should build muscle, and greater mass and grip strength would be expected. The evidence, however, is heterogeneous and phenotype- and BMI-dependent. Absolute grip strength and total lean mass are often greater than in BMI-matched controls, particularly in hyperandrogenic phenotypes, whereas one DXA-based case–control study (68 women with PCOS vs. 60 controls) reported a significantly lower percentage of appendicular skeletal muscle (%ASMM: 23.8% vs. 30.4%, *p* < 0.0001) and found that 53% of PCOS women met its study-specific criteria for sarcopenic obesity [4]. Lean women with PCOS may show an osteosarcopenic pattern even under conventional therapy [5]. The same women can show evidence of a compromised gut barrier—“leaky gut” being a non-technical shorthand for increased intestinal permeability. The association between elevated serum zonulin and PCOS, including its correlation with insulin resistance and the severity of anovulation, has been reported in an age- and BMI-matched cohort [6], and a pilot study of gut microbiome composition and barrier function in women with PCOS likewise documented barrier alterations alongside reproductive and metabolic defects [7]. For a long time, these two observations—increased intestinal permeability on one side, compromised muscle on the other—were regarded as independent by-products of the underlying metabolic syndrome rather than links in one chain. Two signalling axes now offer candidate explanations for how the gut and muscle may be connected, and this review treats them as a pair rather than alternatives.

The first axis—LPS–TLR4 inflammation—is generally well established in obesity and metabolic endotoxemia, and its components are demonstrable in PCOS [8] (Figure 1).

When Gram-negative bacteria overgrow in the gut, circulating lipopolysaccharide rises and engages the CD14/TLR4 complex on leukocytes, adipose macrophages, and skeletal myocytes, activating MyD88-dependent NF-κB signalling and the release of TNF-α, IL-6, and IL-1β [9,10]. These cytokines phosphorylate IRS-1 on serine residues, which blocks the Akt–mTORC1–p70S6K pathway that normally drives muscle protein synthesis and, at the same time, switches on the ubiquitin–proteasome and autophagy–lysosome systems that break muscle protein down [11,12]. Most existing work on the PCOS gut–muscle relationship is built on this LPS axis [13,14].

The second axis is newer to the PCOS literature and receives equal weight: bile acid signalling through FXR and TGR5. Targeted serum profiling—currently reported in a preprint that has not undergone peer review—describes a distinct bile acid signature in women with PCOS, characterized by higher cholic acid and an expanded conjugated bile acid pool; because this evidence is preliminary, its correlation findings should be verified against the full text [15]. Peer-reviewed profiling has since documented altered circulating bile acid profiles in PCOS [16] and in lean hyperandrogenic women [17], and bile acids have been evaluated together with fibroblast growth factor 21 as diagnostic candidates [18]. Part of the explanation may lie in the microbiota. Microbial transformation of human bile acids is a well-characterised function of the gut community, with deconjugation by bile salt hydrolase-expressing taxa such as *Bacteroides* species being among the most common reactions [19]. Deconjugation liberates unconjugated species—including deoxycholic acid, a potent endogenous TGR5 agonist—so it reshapes the endogenous FXR/TGR5 ligand pool rather than uniformly weakening it; whether net FXR/TGR5 signalling falls in PCOS has not been demonstrated directly and is treated here as a proposed step requiring validation [19,20]. We hypothesise that a reduction in three signalling outputs follows from the PCOS bile acid signature: loss of FGF19 (fibroblast growth factor 19; its rodent orthologue is FGF15) signalling, reduced GLP-1 release from intestinal L-cells, and reduced IL-22 secretion from group 3 innate lymphoid cells [21] as depicted in Figure 2.

In experimental systems—largely rodent muscle and brown adipose tissue—TGR5 signalling supports insulin responsiveness, energy expenditure, and adaptive responses to exercise, although TGR5 expression in mature human skeletal muscle is low and contested; FXR, whose skeletal-muscle expression is lower still, is believed to act chiefly through hepatic and intestinal pathways [22,23]. Whether these receptors exert direct effects in human PCOS muscle therefore remains to be established.

The two axes are proposed to converge rather than compete. LPS delivers an inflammation-driven attack on muscle, accelerating proteolysis and establishing anabolic resistance at the myocyte. The bile acid axis is proposed to exert a parallel, substrate-derived influence (operating through metabolic ligands rather than inflammatory cytokines), producing insulin resistance independent of cytokines and disrupting the enterohepatic–muscle endocrine loop [20,22]. Both begin at the same point—a dysregulated gut microbiome and a weakened intestinal barrier—and both end, in this model, at the same point: muscle insulin resistance, mitochondrial dysfunction, and the loss of lean mass and grip strength that defines sarcopenic obesity in PCOS. To the best of our knowledge, and based on the literature search described in Section 1.2, no previous review has examined these two pathways together as parallel mechanisms within a single framework for this population. Three goals guide this review. The first is consolidation: gut permeability data (zonulin, LBP, DAO, sCD14, I-FABP, endotoxin) and muscle outcome data (handgrip strength, DXA-derived appendicular skeletal muscle mass, BIA-derived lean body mass) are rarely reported together, and they are brought into one framework here. The second is rebalancing: the bile acid–FXR/TGR5 axis is underrepresented in PCOS gut–muscle narratives, and this review gives it equal mechanistic standing with the LPS–endotoxemia axis, showing how both may engage the same downstream machinery of muscle protein synthesis and degradation. The third is identifying the knowledge gaps that currently prevent meta-analytic synthesis of the gut–muscle relationship in PCOS, as well as proposing prospective study designs that could close them.

### 1.1. Metabolic Features of PCOS

The metabolic feature most central to the gut–muscle axis is insulin resistance, demonstrable in the skeletal muscle of PCOS women even after BMI-matching to controls [24]. The molecular signature in muscle biopsies and cultured muscle cells shows that, due to impaired insulin signalling in skeletal muscle, insulin is less able to activate IRS-1 through tyrosine phosphorylation, while inhibitory serine phosphorylation increases, resulting in reduced PI3K–Akt activation and diminished GLUT4 translocation. Intrinsic and acquired defects in insulin signalling in the skeletal muscle of women with PCOS, including elevated IRS-1 serine phosphorylation in cultured myotubes passaged away from the in vivo hormonal milieu, have been demonstrated directly in primary cell studies [24]. The gut-derived endotoxin → TLR4 → JNK/NF-κB → IRS-1 serine phosphorylation → impaired muscle glucose uptake chain therefore engages molecular machinery that converges with the IRS-1 serine-phosphorylation machinery identified in PCOS muscle [8,24]. The two pathways were characterised in different populations—non-PCOS human myotubes and TLR4-null rodents versus PCOS biopsy and myotube studies—so their convergence is inferred rather than demonstrated; this inference is the premise on which the central framing of this review rests.

Dyslipidemia in PCOS follows an atherogenic pattern that extends beyond LDL concentration: the LDL pool shifts toward small, dense particles (sdLDL), which are more resistant to hepatic clearance, more susceptible to oxidation, and more atherogenic than large buoyant LDL. sdLDL elevation has been documented in PCOS cohorts alongside hypertriacylglycerolemia and reduced HDL-cholesterol—the classical atherogenic lipoprotein phenotype—with the shift amplified mainly in the obese subgroup [3]. Hepatic comorbidity completes the metabolic profile. NAFLD is present in an estimated 30–70% of PCOS women depending on the diagnostic threshold applied, with hyperandrogenism, insulin resistance, and central adiposity acting as additive contributors [25,26,27,28,29]. The chronic low-grade inflammatory state warrants explicit treatment because it links the features above to the muscle phenotype. Circulating TNF-α, IL-6, IL-1β, IL-18, and hsCRP are elevated in PCOS women versus BMI-matched controls, independent of obesity per se [9,30,31]. The connection to muscle is direct here: TNF-α activates NF-κB, which transcriptionally upregulates the E3 ubiquitin ligases MuRF1 and atrogin-1/MAFbx—the central effectors of the ubiquitin–proteasome system that drive myofibrillar breakdown and reduce myofiber cross-sectional area [11]—while IL-6 and IL-1β act in parallel through JAK–STAT and inflammasome signalling [12]. Together these pathways generate the low-grade “inflammaging” milieu that translates the systemic inflammatory state of PCOS into muscle proteolysis.

### 1.2. Literature Search and Review Approach

We searched PubMed/MEDLINE, Scopus, Web of Science, and Google Scholar from inception to October 2025, with an update in March 2026, using combinations of the terms “polycystic ovary syndrome” or “PCOS” with “gut microbiota”, “intestinal permeability”, “zonulin”, “lipopolysaccharide”, “endotoxemia”, “bile acids”, “FXR”, “TGR5”, “short-chain fatty acids”, “skeletal muscle”, “muscle strength”, “sarcopenia”, “insulin resistance”, and “exercise”. Reference lists of retrieved reviews were screened for additional records. Titles and abstracts were screened independently by A.P. and A.K., with disagreements resolved by consensus; no formal quality scoring was applied, consistent with the narrative design of this review. Inclusion was limited to English-language original studies, meta-analyses, and authoritative reviews reporting at least one gut-barrier, bile-acid, inflammatory, or muscle outcome in, or directly relevant to, women with PCOS. Where the PCOS literature was absent, animal and in vitro evidence was admitted to illustrate signalling pathways, and every such extrapolation is explicitly identified as non-human in the text. The novelty claim that no previous review has integrated the LPS and bile-acid axes for PCOS muscle is based on this retrieved literature.

## 2. Gut Barrier Dysfunction in Polycystic Ovary Syndrome

The framework most often invoked to interpret these findings is the Dysbiosis of Gut Microbiota theory, a hypothesis subsequently reviewed in relation to the pathogenesis of the syndrome [32,33]. The core claim is direct: a dietary pattern dominated by saturated fat and refined sugar shifts the gut microbiota toward Gram-negative, mucin-degrading taxa and away from the barrier-protective genera *Akkermansia*, *Bifidobacterium*, and *Faecalibacterium* [34,35]. As mucin-degrading bacteria expand, tight junction integrity weakens, lipopolysaccharide translocates into the portal and systemic circulations, and the resulting low-grade endotoxemia activates NF-κB. The downstream kinase cascade serine-phosphorylates IRS-1, producing the insulin resistance that drives theca-cell androgen excess—the proposed biological bridge from the gut to the hyperandrogenism that defines the syndrome [32,36]. What the framework did not separate is what more recent evidence demands: the gut-derived mediators are not confined to LPS. A second axis operates in parallel—bile acid signalling through the farnesoid X receptor and the Takeda G protein-coupled receptor 5 [20,21,22,23]. The two pathways diverge at the receptor level but are predicted to converge on the same downstream signalling hub in skeletal muscle, the IRS-1–Akt–mTORC1 axis, and they are treated here as complementary candidate mediators of the same gut-derived impairment rather than as alternative explanations.

### 2.1. Intestinal Permeability Alterations

This evidence is based on six biomarkers that index different elements of the barrier: zonulin, intestinal fatty-acid-binding protein, diamine oxidase, lipopolysaccharide-binding protein, soluble CD14 (sCD14), and direct endotoxin [3,6,7]. It should be noted at the outset that all six are indirect surrogate markers; none of the studies cited used a dual-sugar absorption test or comparable functional measure of permeability. The first report in PCOS came from a cohort of 78 women with PCOS and 63 age- and BMI-matched controls, in which serum zonulin was significantly elevated (*p* = 0.022) and correlated with insulin resistance, the insulin sensitivity index, and the severity of anovulation after adjustment for age and BMI [6]. A separate pilot cohort (24 PCOS vs. 19 age- and BMI-matched controls) confirmed the elevation (serum zonulin *p* = 0.006) and reported it alongside altered gut microbiome composition and barrier function associated with the reproductive and metabolic defects of the syndrome [16]. The pattern—zonulin and DAO are reproducibly elevated, with inconsistent changes in LBP and other inflammatory markers—indicates that gut-derived endotoxin exposure may occur intermittently rather than continuously, with a barrier that is metabolically sensitized rather than broadly compromised [6,7]. The simplest reading is that zonulin behaves as a general metabolic-sensitivity marker. Whether zonulin is activated to higher levels in PCOS than in BMI-matched controls because PCOS adds insulin resistance and androgen excess to the adiposity burden is an interpretation; it is not established by the available data.

One important limitation should be considered when interpreting zonulin results. Some commercially available zonulin ELISA kits may measure a related protein called properdin instead of the actual zonulin molecule (the haptoglobin-2 precursor), so antibody validation matters when comparing measurements across studies [37]. An important counterpoint comes from case–control studies that report no difference in fasting barrier markers between PCOS women and BMI-matched controls—for example, the study by Cetin and colleagues which reported no zonulin elevation in PCOS without metabolic syndrome. Such markers are measurable in this population, but their relationship to PCOS status itself remains incompletely resolved [7]. Rather than refuting the barrier hypothesis, these findings shift its emphasis: the PCOS barrier may be functionally competent at rest but fail more readily when challenged by nutrient load. Direct evidence for that interpretation comes from the Martínez-García 2024 macronutrient-challenge study (17 PCOS women, 17 non-hyperandrogenic controls, and 19 men), in which fasting GLP-2 and postprandial LBP were elevated in PCOS, with a PCOS × obesity interaction [38]. The type of meal also mattered: the high-fat meals produced the least favorable response, protein-rich meals produced the most favorable response, and glucose had an intermediate effect [38]. Postprandial or provoked testing is therefore more sensitive than fasting single-timepoint designs.

Three main conclusions can be drawn from these studies. First, barrier dysfunction in PCOS is real but heterogeneous: zonulin and DAO are reproducibly elevated, LBP tends to be elevated, and sCD14 and calprotectin are not [6,7]. Second, the dysfunction is best detected under dietary load rather than at rest. Third, whether the degree of gut barrier dysfunction in PCOS exceeds that of obesity alone is untested, because none of the included studies compared PCOS head-to-head with a non-PCOS obese group—several used BMI-matched rather than obese comparators; the pattern is tentatively consistent with PCOS sitting at the metabolically sensitive end of the barrier spectrum rather than at a uniquely severe extreme [39,40].

### 2.2. Microbiota Composition Shifts and the Bile Acid–FXR/TGR5 Signalling Axis

The most reproducible microbiota alterations in PCOS are reduced alpha diversity, a relative depletion of *Akkermansia* and the Tenericutes phylum, and an expansion of LPS-bearing Gram-negative taxa—with *Bacteroides vulgatus* and *Prevotella* being the most consistently reported examples [41,42,43]. These shifts are present even in normal-weight PCOS women with insulin resistance, so they cannot be dismissed as a secondary consequence of obesity [41]. At the functional level, gut-derived metabolites such as short-chain fatty acids and indole-3-propionic acid are altered in women with PCOS and are associated with dietary intake [42]. The impact of gut microbiota and short-chain fatty acids on the pathogenesis of PCOS, as well as the effect of metformin therapy on both, has been the subject of dedicated investigation [44].

The most informative quantitative dataset to date profiled 69 PCOS women versus 18 controls, showing depletion of beneficial genera (the *Clostridium leptum* group and *Prevotella*) alongside overgrowth of opportunists (*C. perfringens*, *C. difficile*, *Staphylococcus*, *Streptococcus*) in PCOS women; acetate and valerate were reduced to 47% and 54% of control levels, respectively, and lower acetate was associated with higher BMI, insulin resistance, and systemic inflammation [44]. Because SCFAs support gut-barrier integrity and metabolic signalling, their reduction may further contribute to insulin resistance and muscle dysfunction [44,45].

*Bacteroides* species, including *B. vulgatus*, deserve separate attention here because they carry bile salt hydrolase activity and are among the taxa most active in deconjugating bile acids, a well-characterised microbial transformation of the human bile acid pool [19]. Deconjugation of glycodeoxycholic acid liberates deoxycholic acid, a potent endogenous TGR5 agonist, so the net effect of deconjugation on FXR/TGR5 signalling is not a simple loss of agonism; whether signalling is reduced overall in PCOS is a hypothesis requiring direct testing [19,20]. Gut-microbiota-driven alteration of host bile acid metabolism has been demonstrated experimentally in a reproductive-tract disorder (intrahepatic cholestasis of pregnancy) [20], providing a proof of principle that this mechanism can operate in reproductive pathophysiology. If output is reduced, downstream consequences would include reduced GLP-1 and peptide YY release from intestinal L-cells, reduced ileal FXR-mediated FGF19 secretion, and reduced IL-22 production from group 3 innate lymphoid cells [21].The proposed gut bile acid metabolic signalling pathway is shown in Figure 3.

A study comparing 86 women with PCOS and 60 women without PCOS—currently available only as a preprint that has not undergone peer review—found that women with PCOS have a different pattern of bile acids in their blood [15]. The report describes higher cholic acid, an expanded conjugated pool, and an increased conjugated-to-unconjugated ratio; these correlation findings should be verified against the full text given the preprint status. Peer-reviewed corroboration is accruing: circulating bile acid profile characteristics differ between women with PCOS and controls [16]; altered bile acid profiles are associated with hyperandrogenism in lean women with PCOS [17]; and bile acids combined with fibroblast growth factor 21 have been assessed as diagnostic markers in women with PCOS [18]. Three mechanisms are proposed to transmit this disturbance to peripheral metabolism. First, diminished ileal FXR would reduce FGF19 secretion; reduced FGF19 signalling at hepatic FGFR4/β-Klotho would relieve the suppression of gluconeogenesis and impair glycogen repletion [21]. Second, reduced TGR5 activation in skeletal muscle and brown adipose tissue would blunt the cAMP–PKA–D2 cascade (cyclic adenosine monophosphate, protein kinase A, type 2 deiodinase) that drives type 2 deiodinase activity, mitochondrial oxidative phosphorylation, and insulin-stimulated glucose uptake [22,23]. Third, loss of FGF19 and TGR5 support for pancreatic β-cells would accelerate the trajectory toward β-cell exhaustion [22]. The aggregate effect, if confirmed, suggests that altered bile acid signalling in PCOS may contribute to muscle insulin resistance. Because the bile acid pathway carries such potential metabolic weight for muscle protein synthesis, mitochondrial respiration, and tissue regeneration in experimental systems, it has moved to the foreground of PCOS gut–muscle research alongside the barrier and LPS targets that dominated the earlier literature [21,22,23].

### 2.3. Systemic Inflammation: The Convergent Endotoxemia and Bile Acid Framework

The systemic inflammatory state of PCOS is proposed to emerge at the intersection of the two pathways. The first pathway is the LPS–TLR4 inflammatory pathway. Gut barrier dysfunction allows lipopolysaccharide from Gram-negative bacteria to enter the circulation. LPS binds lipopolysaccharide-binding protein and is then transferred to CD14 and TLR4 receptors on immune cells, liver Kupffer cells, adipose macrophages, and skeletal muscle cells. TLR4 activates NF-κB and AP-1, which increases the production of TNF-α, IL-6, IL-1β, and CRP [12,46]. The experimental foundation in rodent models of diet-induced obesity showed that a high-fat diet increases circulating LPS and that chronic low-dose LPS exposure can induce obesity and insulin resistance even without dietary changes [36,47,48]. Within muscle specifically, LPS–TLR4 signalling reduces insulin-stimulated phosphorylation of IRS-1, Akt, and AS160 [8,49,50,51], upregulates the E3 ubiquitin ligases MuRF1 and atrogin-1/MAFbx that drive ubiquitin-mediated proteolysis [10,11], and impairs oxidative phosphorylation by destabilizing electron-transport-chain assembly [52]. These features have been documented in PCOS women, where elevated TNF-α, IL-6, IL-1β, IL-18, and hsCRP are found compared to BMI-matched controls, independent of obesity [9,30,31]. The second pathway—the bile acid pathway—is proposed to deliver the complementary, substrate-derived signal. Reduced TGR5/PKA signalling in skeletal myocytes and reduced FGF19 signalling at the liver would drive insulin resistance through mechanisms that are independent of inflammation and proceed in parallel rather than downstream of NF-κB activation [21,22,23]. Because the two pathways begin in the gut and are proposed to converge on skeletal muscle, the LPS axis is proposed to deliver the inflammatory strike and the bile acid signature with substrate-level restraint. Together they form the conceptual bridge that links the gut barrier literature to the muscle outcome literature.

## 3. Skeletal Muscle Impairment in Polycystic Ovary Syndrome

### 3.1. Phenotype Heterogeneity and the Anabolic Paradox

Under the Rotterdam criteria, phenotype A combines hyperandrogenism, ovulatory dysfunction, and polycystic ovarian morphology; phenotype B combines hyperandrogenism and ovulatory dysfunction without polycystic morphology; phenotype C combines hyperandrogenism and polycystic morphology with preserved ovulation; and phenotype D comprises ovulatory dysfunction and polycystic morphology without hyperandrogenism. These subphenotypes differ in androgenic and metabolic burden, which may translate into differing muscle characteristics; pooling them can obscure phenotype-specific effects [53]. Throughout this review, “sarcopenic obesity”, “sarcopenia”, and “reduced muscle quality” are used strictly as reported by each source and are not treated as interchangeable; the definition applied is stated wherever a prevalence figure is cited [4].

The dominant interpretation in the literature is, on first inspection, that PCOS women often show greater handgrip strength and higher total lean mass than BMI-matched controls, a finding generally consistent with hyperandrogenic anabolism. One cohort study of 70 women with PCOS and 93 controls found greater dominant-arm lean mass and stronger handgrip strength in women with PCOS, especially in the hyperandrogenic A and B subgroups [54]. The association is not universal, however; in some cohort studies, circulating testosterone shows no association with lean mass, and lean PCOS women can display lower lean mass than BMI-matched controls despite higher testosterone. A meta-analysis of 45 studies including 3676 women found that women with PCOS had slightly higher total lean mass than controls, that this difference was mainly associated with BMI rather than hyperandrogenism or insulin resistance, that women with BMI 25 or higher gained about 1.6 kg of lean mass, and that lean women with PCOS tended to have less lean mass than controls [53].

The DXA-based case–control study (68 PCOS vs. 60 controls) reported significantly lower appendicular skeletal muscle mass as a percentage of body mass (%ASMM) in women with PCOS compared with controls (23.8% vs. 30.4%, *p* < 0.0001) [4]. In that cohort, 53% of women with PCOS met the study-specific criteria for sarcopenic obesity, defined as %ASMM more than two standard deviations below the control mean combined with body fat > 35%—a definition that is not identical to EWGSOP2, FNIH, or ESPEN/EASO criteria, so the prevalence is not directly generalisable to all women with PCOS. In addition, %ASMM was inversely correlated with hsCRP (r = −0.608, *p* < 0.0001) and HOMA-IR (r = −0.409, *p* < 0.01) [4]. A pilot study in lean PCOS women already on metformin and oral contraceptives (17 PCOS vs. 17 controls) showed reduced muscle mass, glycogen, protein, and bone mineral content together with expanded extracellular water (all *p* ≤ 0.021)—an osteosarcopenic phenotype present even on conventional therapy [5].

Two important points are to be noted here. First, the direction of effect depends on the phenotype and BMI stratum: lean women and women with a high metabolic burden show the clearest muscle impairment. Second, the more precise concept in PCOS is not lean mass per se but muscle quality—denoted as function per unit of lean mass. Total mass is often higher, but normalized functional outcomes are more reproducibly reduced in lean and high-metabolic-burden subgroups [4,53]. Because consensus definitions differ (Baumgartner appendicular lean cut-offs, FNIH, EWGSOP2, and ESPEN/EASO), prevalence varies with the definition applied. The phrase “PCOS is a risk factor for sarcopenic obesity” is therefore better reframed, in this review, as “PCOS is a risk factor for reduced muscle quality and elevated sarcopenic-obesity prevalence in the lean and high-metabolic-burden subgroups,” a phrasing that more accurately reflects the heterogeneity above [4,53].

### 3.2. Insulin Sensitivity in Skeletal Myocytes

PCOS myocytes display an intrinsic, BMI-independent insulin-resistance phenotype that goes beyond the systemic metabolic-syndrome pattern [24]. Intrinsic and acquired defects in insulin signalling in the skeletal muscle of women with PCOS—including impaired insulin-stimulated IRS-1 tyrosine phosphorylation with competing serine phosphorylation, as well as reduced downstream PI3K–Akt activation and glucose transport—have been demonstrated in primary muscle cells and biopsies [24]. A useful tissue-specific distinction is that, in PCOS, skeletal muscle displays impaired insulin responsiveness, while adipocytes display impaired insulin sensitivity with preserved responsiveness [24]. Bile acid signalling may contribute to this muscle-specific defect. Normally TGR5 activation in myocytes would support insulin-stimulated glucose uptake and oxidative phosphorylation [22,23]. Distortion of the PCOS bile acid pool is hypothesised to lower TGR5 ligand availability at L-cells, reduce FXR-mediated FGF19 secretion, and cause loss of FGF19/TGR5 support for pancreatic β-cells, removing the intrinsic sensitizing signal by a route that is mechanistically independent of the inflammatory IRS-1 serine-phosphorylation cascade [22,23]. A separate, more subtle impairment is that PCOS muscle may respond suboptimally to exercise training, with incomplete translation of training into metabolic adaptation at the myocyte [3,55]; direct comparative trials of training responses in PCOS remain limited. Hyperandrogenism sits at the centre of these data, but its causal weight in humans remains modest. In the DHEA-induced mouse model, oxidative stress in skeletal muscle has been shown to contribute to insulin resistance in PCOS, and hyperandrogenism-driven mTORC1 activation with suppressed autophagy has also been implicated in muscle insulin resistance in the same model [56]. Human biopsy data, by contrast, find little evidence that hyperandrogenism by itself drives insulin resistance in patients, and the discrepancy reflects the gap between supraphysiologic androgen load in rodent PCOS models and the ambient physiologic androgen elevations typical in chronic clinical PCOS. Overall, hyperandrogenism appears to be one contributing mechanism rather than the sole cause of insulin resistance in PCOS.

### 3.3. Mitochondrial Dysfunction Markers

Mitochondrial dysfunction in PCOS muscle is documented at multiple levels, including reduced oxidative-phosphorylation enzyme expression, reduced mtDNA content, and ultrastructural abnormalities on biopsy [57,58]. Reduced expression of nuclear-encoded genes involved in mitochondrial oxidative metabolism has been demonstrated in skeletal muscle of insulin-resistant women with PCOS [58]. Most quantitative claims come from the DHEA (dehydroepiandrosterone)-induced rodent model discussed earlier, in which hyperandrogenism-driven mTORC1 activation and autophagy suppression cause dysfunctional mitochondrial accumulation [56,58]. Human data are limited and inconsistent: one human study reported no significant reduction in muscle mitochondrial respiration or content in women with PCOS, whereas the gene-expression data indicate reduced oxidative-metabolism capacity [57,59]. Our reading that mitochondrial dysfunction may be more pronounced in hyperandrogenic and metabolically severe animal models than in patients is an interpretation of a small and discordant literature [56,57]. The most directly relevant human evidence for the muscle side of this review is the proteomic analysis of skeletal muscle from women with PCOS, which reported decreased Type I (oxidative) fibres and ectopic fat accumulation [60]. The consequence is a shift toward a more glycolytic, lower-quality fibre distribution, and this can occur even when total lean mass is preserved or elevated [4,60].

### 3.4. Sarcopenia Risk Patterns and Functional Markers

Absolute isometric grip strength and 1-RM (one-repetition maximum) are generally greater in PCOS, with the effect driven by BMI and hyperandrogenism compared to non-PCOS women [53,54,61]. But this is not true for all aspects of muscle function: a range of functional markers that depend on Type II fibre recruitment and oxidative capacity—such as peak power output, rate of force development, fatigue resistance, and BMI-normalized strength—may be poorer. This pattern is consistent with the reduced Type I fibre density documented by proteomics [60]. The apparent conflict between absolute and quality measures therefore resolves rather than dissipates: PCOS does not uniformly impair all strength categories. Importantly, the skeletal muscle in PCOS appears responsive to resistance training, and this responsiveness has been examined in a randomised controlled trial of resistance training in non-obese women with PCOS [55] as well as in a pilot feasibility trial without formal power for efficacy [62]. In the pilot trial, upper-body strength showed no significant between-group difference (*p* = 0.06) and lower-body strength rose significantly (*p* = 0.03); these exploratory findings require confirmation in adequately powered trials before they can underwrite the resistance-training component of the strategy developed in Section 4.6 [62].

## 4. A Unified Pathophysiological Mechanism Bridging Gut Barrier Dysfunction and Skeletal Muscle Health in PCOS

### 4.1. The Dual-Axis Convergence

Section 2 summarises the gut-side evidence: PCOS women show elevated zonulin and diamine oxidase, LPS translocation, and a bile acid pool shifted toward an elevated conjugated signature [,6,7,15]. Section 3 summarises the muscle-side evidence: impaired glucose uptake, reduced mTORC1 signalling, fewer Type I fibres, mitochondrial dysfunction, reduced %ASMM in lean and high-metabolic-burden cohorts, and reduced normalized function in non-hyperandrogenic phenotypes [4,24,60]. These findings are not independent. Two parallel candidate mediator axes connect these streams. Both are proposed to share the same downstream hub—IRS-1→Akt→mTORC1. Both arise from the same upstream disturbance, gut dysbiosis and barrier dysregulation, and both are proposed to converge on the same muscle phenotype. This model fits with the gut–muscle axis that has already been described in aging, but it extends that concept to PCOS [11,23]. In PCOS, gut barrier dysfunction and altered bile acid signalling are hypothesised to act together to influence skeletal muscle metabolism and structure. Thus, the gut and muscle changes seen in PCOS should be viewed, for the purposes of this review, as candidate components of a single pathophysiological process rather than as separate abnormalities.

### 4.2. The Unified Pathophysiological Mechanism

Both the intestinal epithelium and the myocyte express TLR4, both rely on IRS-1 → Akt → mTOR signalling, and both respond to the same cytokines; evidence for the epithelial half is drawn from studies of barrier regulation, and separate muscle-specific evidence is cited for the myocyte half of this statement [8,24,63]. The proposed mechanism integrates these streams in five steps, each presented as a hypothesis to be tested, with the bile acid axis acting as a parallel substrate-derived input.

Step 1—Tight junction loosening and bile acid microbiota disruption. In PCOS, the intestinal barrier is disrupted as zonulin loosens the claudin/occludin/ZO-1 scaffold (serum zonulin *p* = 0.022) [6], with gut microbiome composition and barrier function altered in a PCOS pilot cohort [16]. Microbiota shifts toward Gram-negative, mucin-degrading taxa with displacement of *Akkermansia* and other SCFA-producing organisms, which raises mucosal LPS and shrinks the SCFA pool that fuels colonocyte tight-junction resynthesis [44,64,65]. In parallel, *Bacteroides* species deconjugate bile acids such as glycodeoxycholic acid through bile salt hydrolase activity [19]. Deconjugation reshapes—rather than uniformly depletes—the FXR/TGR5 ligand pool, because it liberates deoxycholic acid, a potent endogenous TGR5 agonist; whether net FXR/TGR5 signalling falls in PCOS is unproven [19,20].

Step 2—Pathogen-associated molecular pattern translocation and bile acid pool distortion. Damaged tight junctions permit lipopolysaccharide to enter the portal circulation. LPS is bound by lipopolysaccharide-binding protein and transferred to cluster of differentiation 14, which presents it to Toll-like receptor 4 on intestinal epithelial cells, macrophages, and skeletal muscle (LBP trend, *p* = 0.053) [38]. The experimental foundation in rodent models of diet-induced obesity shows that high-fat feeding raises plasma LPS two- to threefold, and low-dose LPS infusion reproduces diet-induced obesity strictly as reported as well as insulin resistance [36,47,48]. Postprandial dietary challenges increase circulating endotoxin and TLR expression, explaining why detection in PCOS is intermittent [38]. Ligand depletion would propagate in parallel: reduced ileal FXR diminishes FGF19 (fibroblast growth factor 19; rodent orthologue FGF15) secretion from ileal enterocytes, removing the hepatic FGFR4/β-Klotho brake on gluconeogenesis; reduced TGR5 diminishes GLP-1/PYY, leading to impaired insulin secretion, reduced satiety signalling, and increased postprandial glucose; and reduced IL-22 compromises antimicrobial peptide production, amplifying Step 1 [21].

Step 3—Innate immune activation and FXR/TGR5 signalling. The LPS–LBP–sCD14–TLR4 complex activates myeloid differentiation primary response 88-dependent NF-κB in circulation, adipose macrophages, and muscle-resident macrophages [36,66]. PCOS women show elevated TNF-α, IL-6, IL-1β, and CRP versus BMI-matched controls [9,10]. Muscle TLR4 promotes intramuscular inflammation even when systemic cytokine levels are not markedly elevated [8,51]. At the same time, impaired bile acid signalling is proposed to weaken metabolic regulation further. Reduced TGR5 in myocytes would decrease cyclic adenosine monophosphate (cAMP)–protein kinase A signalling and type 2 iodothyronine deiodinase (D2), reducing mitochondrial oxidative phosphorylation and insulin-stimulated glucose uptake. Reduced activation of the farnesoid X receptor in the liver would decrease fibroblast growth factor 19 signalling at hepatic FGFR4/β-Klotho—with FGF19 being ileum-derived—leading to increased gluconeogenesis and ectopic lipid accumulation [22,23]. Both axes are therefore proposed to reach Step 4 from different directions: cytokine-driven JNK/IKKβ versus reduced TGR5/PKA and FGF19 signalling.

Step 4—IRS-1 serine phosphorylation (the central bridge). The inflammatory and bile-acid pathways described in the preceding steps are proposed to converge on a common insulin receptor substrate-1. Under normal physiological conditions, insulin binding induces tyrosine phosphorylation of IRS-1, enabling activation of the PI3K–Akt pathway, GLUT4 translocation, glycogen synthesis, and anabolic signalling [67]. In PCOS, chronic exposure to TNF-α and IL-6 activates the stress kinases JNK and IKKβ, which phosphorylate IRS-1 at the inhibitory serine residue Ser307 (rodent numbering; the human orthologue is Ser312) [8,24]. The human orthologue Ser312 is constitutively phosphorylated in cultured myotubes from women with PCOS [24]. PCOS muscle shows reduced insulin-stimulated glucose uptake independent of BMI and fasting glucose [24]. The blunted Akt arm limits mTORC1–p70S6K, establishing anabolic resistance before any histological change in lean mass [4,24]. The bile acid axis is proposed to converge on the same hub: reduced TGR5–cAMP–PKA signalling would lower AS160 phosphorylation and GLUT4 mobilization independently of inflammation; reduced FGF19 would elevate hepatic glucose output; and loss of bile-acid-driven oxidative phosphorylation would lower the ATP charge needed for Akt activation [21,22,23]. Both inputs are proposed to converge on the same output.

Step 5—Impaired muscle protein homeostasis. With mTORC1–p70S6K suppressed, protein synthesis falls. The inflammatory environment activates the ubiquitin–proteasome system (MuRF1, atrogin-1/MAFbx) and the autophagy–lysosome system [11,12]. Myostatin upregulation further blunts IRS-1/PI3K/Akt signalling. Thus, the earliest consequence of insulin resistance in PCOS muscle may not be overt atrophy, but an impaired ability to synthesize protein in response to insulin, amino acids, and mechanical loading. Failed insulin signalling exhausts glycogen and impairs oxidative efficiency [24]. The bile acid arm is proposed to compound this through reduced oxidative phosphorylation capacity, elevated glucose substrate load, and loss of mitochondrial biogenesis [22,57].

### 4.3. Potential PCOS-Specific Amplifiers of the Dual-Axis Mechanism

The two pathways may be reinforced by four syndrome-specific amplifiers that could simultaneously increase gut permeability, endotoxemia, and bile acid signalling disturbance. These amplifiers are proposed as plausible but unverified extensions of the model, because the underlying links have not each been demonstrated in PCOS cohorts.

Hyperandrogenism-to-gut axis (proposed). Elevated free testosterone may modify enterohepatic bile acid recirculation and permit an LPS-bearing *Prevotella*-dominant microbiota [44,68,69]; sex differences in gut microbiota composition and in gonadal steroid metabolism have been documented in humans [70,71]. However, fasting serum endotoxin did not differ between PCOS women and BMI-matched controls in the cohort cited above [38], and we therefore do not claim that PCOS is associated with two-fold higher circulating endotoxin. The quantitative claim is withdrawn pending primary evidence. Any reduction in effective FXR and TGR5 signalling would, in turn, impair FGF19-mediated metabolic regulation [22,23].

Loss of progesterone (proposed). Anovulation reduces progesterone levels. Progesterone has been reported to upregulate claudin-1 and claudin-4, and its loss could therefore increase intestinal permeability, facilitating greater translocation of LPS and other microbial products. Progesterone deficiency could also reduce gallbladder-mediated bile-acid delivery to the ileum, diminishing ileal FXR activation and downstream FGF19 secretion. Neither step has been tested directly in PCOS; the molecular claims are cited to general reviews of sex hormones and the microbiome [68,69] and require primary sources [22,23].

Adipose macrophage reservoir (proposed). Visceral adiposity is enriched in PCOS. It may act as a TNF-α/IL-6 production organ that re-amplifies Step 3 and perturbs enterohepatic flux [9,10].

Loss of exercise-induced anti-inflammatory tone (proposed). Reduced habitual activity and reduced exercise-induced IL-10 release would remove the counter-inflammatory brake on TLR4 signalling [72,73], and loss of exercise-driven TGR5 activation would attenuate muscle oxidative capacity [22,72].

### 4.4. Resulting Muscle Phenotype

The predicted convergent endpoint matches the documented PCOS phenotype. Women with PCOS have significantly lower appendicular skeletal muscle (%ASMM): 23.8% versus 30.4% (*p* < 0.0001) in a single DXA-based case–control study, in which 53% of PCOS women met the study-specific sarcopenic-obesity criteria and %ASMM correlated inversely with HOMA-IR (r = −0.409) and hsCRP (r = −0.608) [4]. The proteomic substrate sits beneath these numbers: reduced oxidative Type I fibres with ectopic fat accumulation in PCOS muscle [61]. The contrary findings of greater absolute strength are best understood as the hyperandrogen-dominated A/B subgroup, where androgen-driven anabolic signalling may temporarily offset the suppressive effects of inflammation and bile-acid dysregulation [55]. In the pooled analysis of 45 studies, women with BMI ≥ 25 showed the anabolic pattern of higher lean mass (about 1.6 kg), while lean subgroups showed reduced muscle performance [54]. Phenotype-stratified analyses are consistent with this pattern: only the A/B phenotypes show elevated appendicular lean mass [54,55]. The phenotype- and BMI-stratified pattern is therefore compatible with the model rather than a contradiction of it; observational data of this kind cannot confirm it.

### 4.5. Summary Position

The dual-axis model—LPS–TLR4–inflammation–IRS-1 combined with bile acid–FXR/TGR5—would simultaneously account for (i) elevated zonulin and gut barrier alterations [6,7]; (ii) the altered bile acid signature [15,16]; (iii) elevated TNF-α, IL-6, and HOMA-IR [9,10,24]; (iv) reduced lean mass, %ASMM, and strength [4,53].This is demonstrated in Figure 4.

The key next step is to test this model in a single cohort by measuring gut permeability markers, bile acid profiles, inflammatory mediators, and muscle outcomes simultaneously. If the model is correct, bile acid signalling markers should predict muscle mass and strength independently of inflammatory markers.

### 4.6. Therapeutic Implications of Dual-Axis Targeting

No trial has yet compared single-axis with dual-axis targeting in PCOS, and existing single-target data do not establish that current approaches are insufficient; the dual-axis model instead provides a rationale for testing combined strategies. The model rationalises the following intervention classes as given in Table 1.

Single-axis approaches have not normalised all metabolic or muscle endpoints in available studies. Metformin improves insulin sensitivity and modifies the gut microbiota and SCFA profile in PCOS [44], but no study has tested its effect on the bile acid pool in PCOS, and the osteosarcopenic profile observed in women already receiving metformin plus oral contraceptives comes from a cross-sectional pilot and cannot be attributed to treatment [5]. A clinically meaningful advance would likely require engaging both arms concurrently, but this remains a testable prediction rather than an established requirement [22,74]. Phenotype targeting follows from the model: the non-hyperandrogenic phenotype D subgroup lacks androgen-driven anabolic compensation and may be a high-yield target, while hyperandrogenic A/B phenotypes may ultimately require combined dual-axis and androgen-modulating strategies; both remain testable predictions, because phenotype-stratified intervention data are lacking [4,53]. Dietary patterning modulates the same axes: Mediterranean diet adherence is inversely associated with testosterone and CRP in PCOS, while ketogenic and low-glycemic approaches have been evaluated for inflammatory and glycemic endpoints [82,83,84].

### 4.7. Experimental Designs to Test the Central Hypothesis

All five designs below are proposed future studies; none has been conducted.

Direct-effect cross-sectional study. A prospective cross-sectional cohort would measure, simultaneously, the six gut-barrier biomarkers, the bile acid pool signature plus FGF19/GLP-1, the inflammatory panel, and standardized muscle outcomes (%ASMM by DXA, bilateral grip, BMI-normalized power, MRI mid-thigh area, fibre-type proteomics). The design should specify %ASMM as the single primary muscle outcome and pre-specify which associations are confirmatory (e.g., %ASMM vs. zonulin and vs. cholic acid) versus exploratory; multiplicity should be controlled and confounders pre-registered: age, BMI, PCOS phenotype, metformin and oral-contraceptive use, diet, physical activity, menstrual status, and insulin resistance [4,7,38,53]. A finding that the bile acid–muscle association remains significant after adjusting for inflammation would be consistent with the hypothesis; a cross-sectional association cannot confirm causality.

Mendelian randomization. Instrumental-variable analysis would focus on cholic acid and TGR5-ligand signatures, using existing microbiome–SCFA Mendelian randomisation frameworks [85]. Genetic instruments for circulating bile acids are currently weak, and the analysis should name the GWAS-derived instruments used and formally assess the exclusion-restriction (no-pleiotropy) assumptions; results would remain hypothesis-generating.

Prospective longitudinal cohort. Newly diagnosed, phenotype-stratified participants would undergo serial dual-axis panels over 24 months. The key question is whether changes at 6–12 months predict changes in %ASMM and grip strength at 12–24 months [4,5,53].

Mechanistic intervention RCT. A combined dual-axis intervention (e.g., synbiotic plus polyphenol-rich diet) would be compared against single-arm and placebo groups. The primary outcome would be change in %ASMM at 6 months, with secondary outcomes of phenotype-stratified grip, all four biomarker panels, and the fibre-type panel [56,62,80].

Preclinical validation. In the DHEA-induced PCOS mouse model, a dual-axis intervention would be tested for reversal of impaired muscle protein homeostasis in parallel with normalization of gut-barrier and bile acid panels [56,86]. Rodent models already show that faecal microbiota and *Lactobacillus* transplantation restores estrous cycles, reduces androgens, and normalizes ovarian morphology [78,87]. These designs would generate one integrated PCOS signature: a PCOS cohort with simultaneously elevated barrier and bile acid markers, elevated inflammatory markers, reduced %ASMM and Type I fibre density, and reduced normalized grip across phenotypes [6,15,60].

### 4.8. Limitations of the Current Evidence and Review

This review has limitations that qualify its conclusions. First, it is narrative rather than systematic; selection bias is possible, and although contrary evidence was actively sought, it was not exhaustively quantified. Second, synthesised cohorts applied heterogeneous diagnostic criteria and differed in age, BMI, phenotype, medication, and ethnicity. Third, much of the mechanistic chain is extrapolated from non-PCOS human tissue, rodent models, and in vitro systems. Fourth, no study has yet measured gut permeability, bile acid profiles, inflammatory mediators, and muscle outcomes in the same PCOS cohort, so the central convergence remains a proposed model. Fifth, the permeability biomarkers reviewed are indirect surrogates; no included study used a functional dual-sugar test [6,7]. Sixth, sarcopenia definitions varied across studies, and the 53% prevalence derives from one study-specific definition [4]. Seventh, the available designs do not permit causal inference. Eighth, the therapeutic implications in Section 4.6 are testable predictions rather than established indications, and clinical evidence for the proposed interventions—particularly faecal microbiota transplantation and FXR/TGR5-targeted agents—is limited or absent. Finally, the model is advanced for reproductive-aged women, consistent with the synthesised cohorts [53], and some foundational evidence is preliminary—most importantly the preprint reporting the PCOS serum bile acid signature—and should be consolidated as peer-reviewed data accrue [15].

## 5. Conclusions

This review has integrated two previously siloed evidence streams in PCOS—the gut-barrier–endotoxemia stream and the bile acid–FXR/TGR5 stream—and proposes that they converge on a single shared signalling hub, IRS-1 → PI3K → Akt → mTORC1, potentially producing the sarcopenic-obesity phenotype documented in the disease but not adequately explained by the LPS axis alone. The five-step dual-axis mechanism extends, rather than replaces, the prior LPS model by incorporating the substrate-derived bile acid mediator now described in PCOS serum and identified as a muscle metabolic regulator in experimental systems. The four proposed amplifiers, if confirmed, would act on both axes concurrently. If the specified study designs confirm the model, therapeutic strategies in PCOS might reasonably move toward coordinated, dual-axis approaches that address gut-barrier integrity, bile acid signalling, and skeletal muscle quality simultaneously.

## Figures and Tables

**Figure 1:**
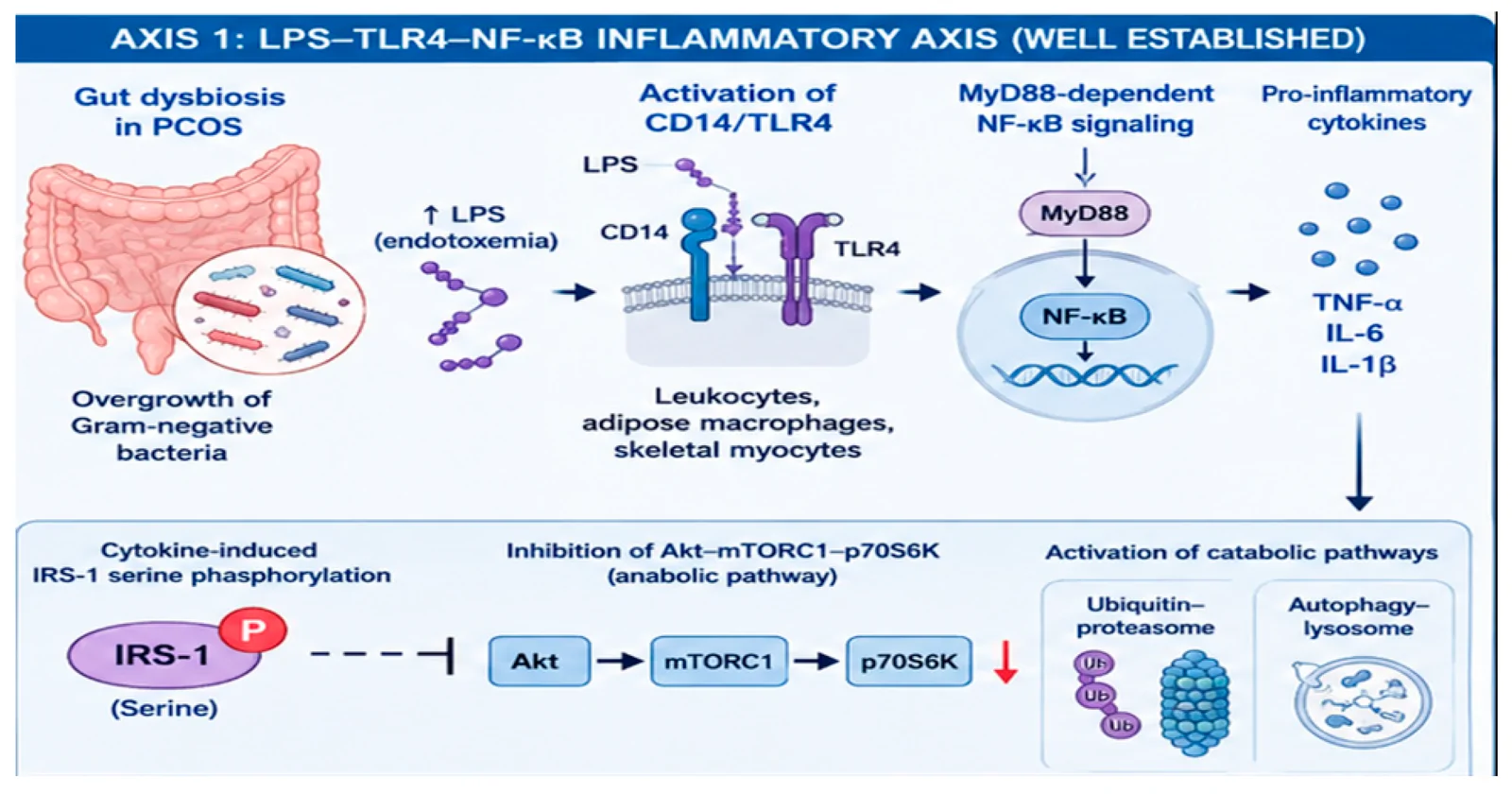
LPS–TLR4–NF-κB inflammatory axis linking gut dysbiosis to skeletal muscle dysfunction in PCOS. Solid arrows denote relationships demonstrated in women with PCOS. Abbreviations: LPS, lipopolysaccharide; TLR4, Toll-like receptor 4; NF-κB, nuclear factor kappa B; MyD88, myeloid differentiation primary response 88; IRS-1, insulin receptor substrate-1; Akt, protein kinase B; mTORC1, mechanistic target of rapamycin complex 1; p70S6K, p70 ribosomal S6 kinase; TNF-α, tumor necrosis factor-alpha; IL, interleukin.

**Figure 2:**
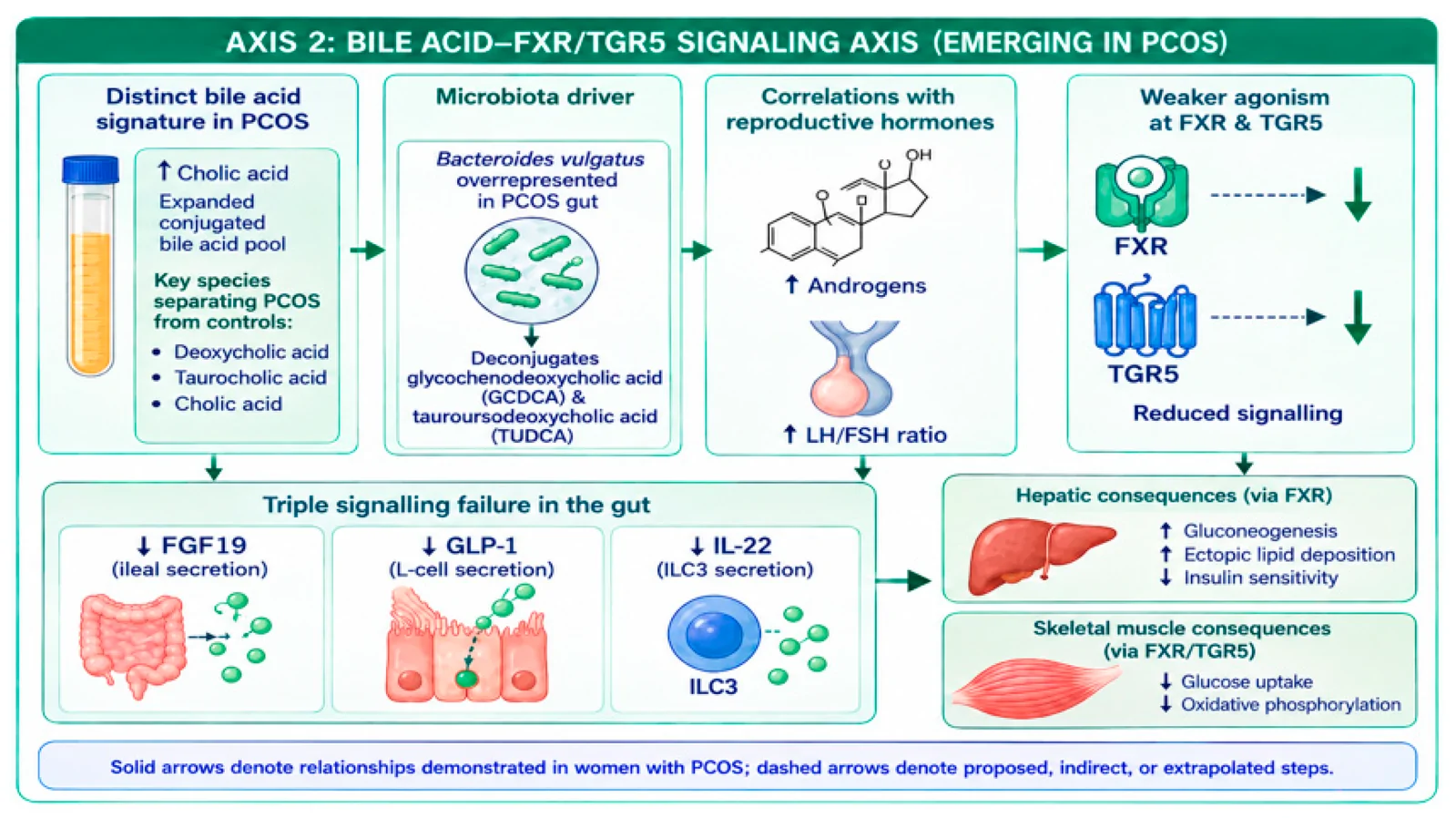
Proposed bile acid–FXR/TGR5 signalling axis linking altered bile acid metabolism to predicted hepatic and skeletal muscle consequences in PCOS. The figure depicts the consequences of reduced FXR/TGR5 signalling, including increased hepatic gluconeogenesis, increased ectopic lipid deposition, impaired insulin sensitivity, reduced skeletal-muscle glucose uptake, and reduced oxidative phosphorylation. Solid arrows denote relationships demonstrated in women with PCOS; dashed arrows denote proposed, indirect, or extrapolated steps. Abbreviations: FXR, farnesoid X receptor; TGR5, Takeda G protein-coupled bile acid receptor 1; FGF19, fibroblast growth factor 19 (FGF15 in rodents); GLP-1, glucagon-like peptide-1; IL-22, interleukin-22; ILC3, group 3 innate lymphoid cells; GCDCA, glycochenodeoxycholic acid; TUDCA, tauroursodeoxycholic acid; LH, luteinizing hormone; FSH, follicle-stimulating hormone.

**Figure 3:**
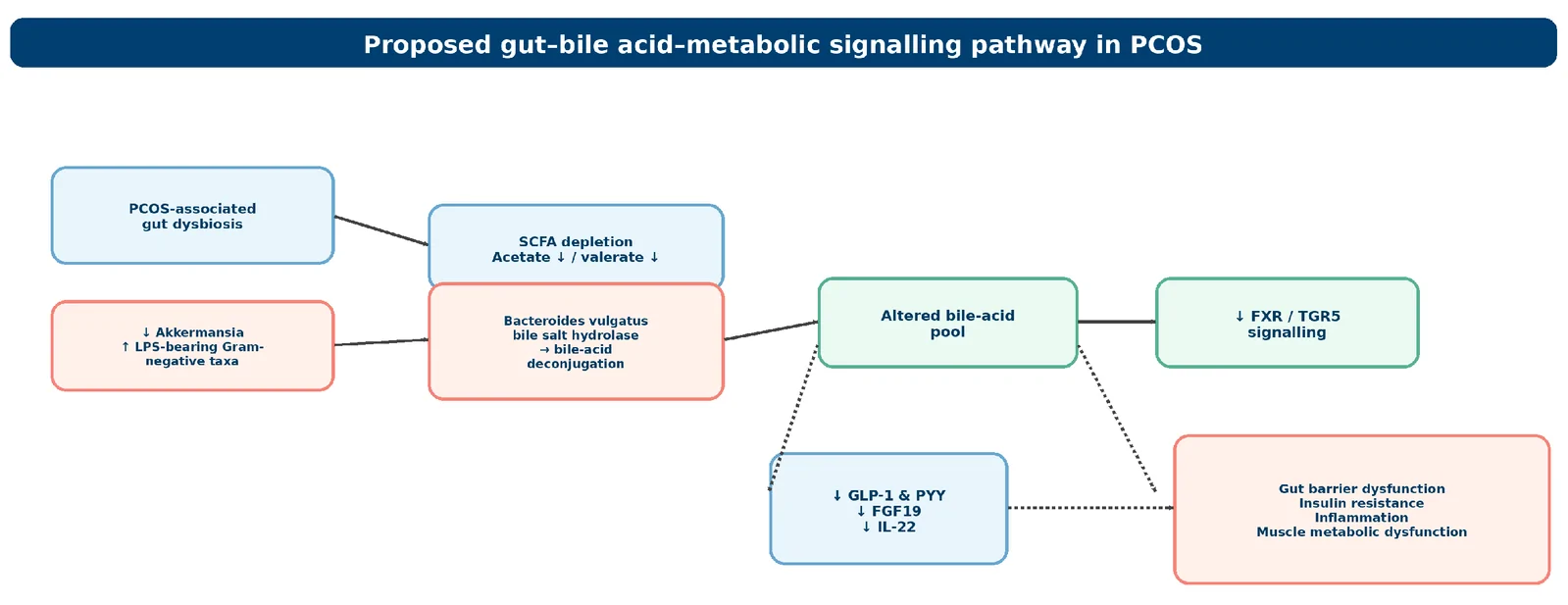
Proposed gut–bile acid–metabolic signalling pathway in PCOS. Solid lines denote relationships demonstrated in women with PCOS; dashed lines denote proposed, indirect, or extrapolated steps. Abbreviations: PCOS, polycystic ovary syndrome; SCFA, short-chain fatty acid; LPS, lipopolysaccharide; FXR, farnesoid X receptor; TGR5, Takeda G-protein-coupled receptor 5; GLP-1, glucagon-like peptide-1; PYY, peptide YY; FGF19, fibroblast growth factor 19 (FGF15 in rodents); IL-22, interleukin-22.

**Figure 4:**
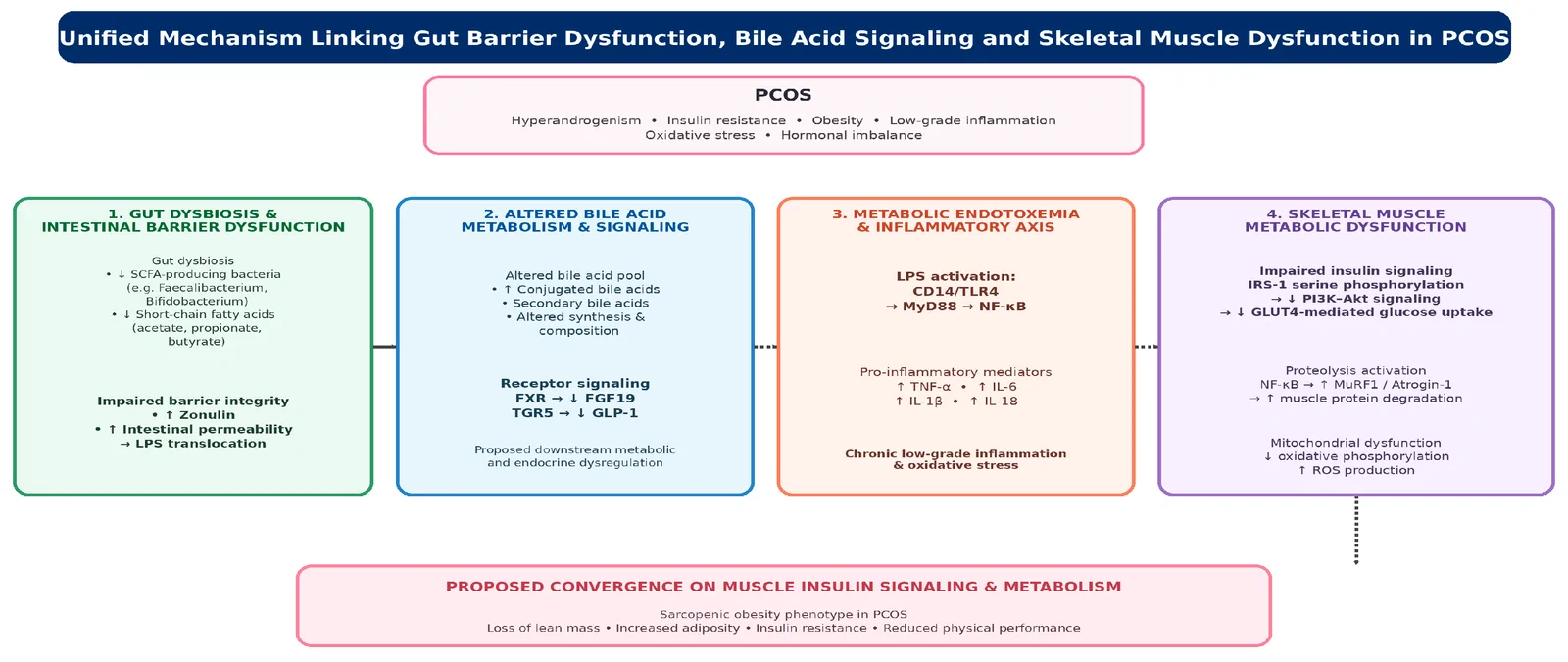
Proposed unified pathophysiological mechanism linking gut barrier dysfunction, bile acid signalling, metabolic endotoxaemia, and skeletal muscle dysfunction in PCOS. Solid arrows denote relationships demonstrated in women with PCOS.

**Table 1:** Dual-axis intervention classes in PCOS, with current evidence level.

Class	Axis Engaged	Mechanistic Node	PCOS Evidence	Evidence Level/Current Status
Bile acid pharmacology (FXR/TGR5 agonists, sequestrants)	Bile acid axis	Ligand restoration; FGF19/GLP-1 reconstitution	Bile acids evaluated as PCOS biomarkers [18].Preclinical modulation of FXR/TGR5 signalling [20,22];	Preclinical/mechanistic plausibility only; no PCOS trials. Clinical development of FXR agonists has been complicated by pruritus and hepatic safety signals
TLR4-targeted pharmacology	LPS axis	Receptor/kinase blockade	Rodent TLR4 deletion/inhibition preserves muscle insulin sensitivity [49,50,51]	Non-PCOS preclinical; mechanistic plausibility; no PCOS clinical data
Adjunct/supportive strategies	Both (supportive)	Antioxidant, anti-inflammatory support	Oxidative stress as a contributor to skeletal-muscle insulin resistance in a rodent PCOS model [56]	Preclinical; supportive role only
Microbiome-targeted (synbiotics, prebiotics, polyphenols)	Both axes	Deconjugation modulated; ligand pool and barrier-protective taxa restored	Meta-analysis of 26 randomised controlled trials reporting reductions in weight, BMI, waist circumference, insulin, HOMA-IR, triglycerides, LDL, and testosterone [74]; probiotic/synbiotic RCTs [75,76,77]	Human PCOS RCTs (meta-analysed) for metabolic endpoints; no trial has measured muscle outcomes
Faecal microbiota transplantation	Both axes (proposed)	Donor microbiome engraftment	Animal-model and mechanistic support only [78]	Experimental; no human PCOS RCT; documented safety risk—not currently a PCOS therapy
Energy restriction/low-glycaemic-index diet	Both axes	Postprandial LBP attenuation; metabolic improvement	Restricted-energy low-glycaemic-index diet trial in overweight women with and without PCOS [79]	Human PCOS interventional (metabolic endpoints); intermittent fasting was not tested in the cited study
Lifestyle/exercise	Both axes	IL-10 tone restored; TGR5-mediated oxidative capacity	Randomised controlled trial of resistance training in non-obese women with PCOS [56]; combined-training RCT [80]; high-intensity interval and strength training pilot in PCOS [62,81]	Human PCOS RCTs and pilot trials; small effect sizes in the pilot studies

## Data Availability

No new data were created or analyzed in this study. Data sharing is not applicable to this article.
